# Modeling and Implementation of Synchronization for Large-Aperture Electromagnetic MEMS Mirrors

**DOI:** 10.3390/mi16030268

**Published:** 2025-02-26

**Authors:** Fahu Xu, Lingxiao Zhao

**Affiliations:** 1 College of Mechanical and Electronic Engineering, Northwest A&F University, Yangling 712100, China; 2 Tianjin Jinhang Institute of Computing Technology, Tianjin 300308, China

**Keywords:** electromagnetic MEMS mirror, synchronization, modeling

## Abstract

MEMS-based LiDAR has showcased extensive application potential in the autonomous driving sector, attributed to its cost-effectiveness, compactness, and seamless integration capabilities. However, MEMS LiDAR suffers from a short detection range, due to the small receiving aperture of the MEMS mirror. Our early study attempted to increase the detection range of MEMS LiDAR with a semi-coaxial design. In this paper, we further investigate the synchronization method for large-aperture electromagnetic MEMS mirrors, in which a synchronous motion transfer model of electromagnetic MEMS mirrors is constructed. The results of the simulations and experiments demonstrate that two electromagnetic MEMS mirrors are synchronous with an aperture of 60 ${\pi \;\mathrm{mm}}^{2}$, FoV of 60°, and scanning frequency of 220 Hz. The entire synchronization process of the electromagnetic MEMS mirrors is completed within 10 s, which verifies the feasibility of synchronizing large-aperture electromagnetic MEMS mirrors to increase the detection range of MEMS LiDAR.

## 1. Introduction

Micro-electro-mechanical system (MEMS)-based LiDAR refers to a miniature LiDAR sensor, which combines MEMS mirror scanning technology and laser ranging technology, to achieve three-dimensional environmental perception [1,2,3]. The working principle of MEMS LiDAR involves the reflection of the laser beam emitted by the laser source using a MEMS mirror to quickly scan the surrounding environment, collecting the reflected light signals, and determining the distance, speed, and shape of target objects by calculating the time of flight (ToF) or phase changes of the light signals [4,5,6]. Benefiting from the miniaturization of the MEMS mirror, MEMS LiDAR has the advantages of small size, light weight, low cost, and ease of integration, demonstrating distinct technical advantages in the field of autonomous driving when contrasted with conventional mechanical LiDAR [7,8,9].

**Figure 1:**
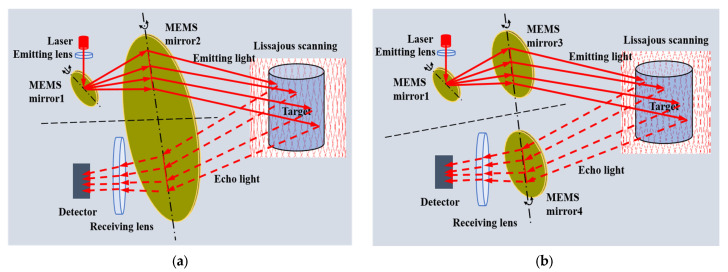
(**a**) Semi-coaxial design with an ultra-large-aperture MEMS mirror; (**b**) Semi-coaxial design with two synchronous MEMS mirrors.

However, MEMS LiDAR usually suffers from a short detection range due to the small receiving aperture of the MEMS mirror [10]. Additionally, a design trade-off exists between the scanning speed, size, and tilt angle of the MEMS mirror [11]. It faces significant challenges in designing and manufacturing a larger-sized MEMS mirror, and the sizes of existing MEMS mirrors are generally a few millimeters [12]. In our early study, we proposed a semi-coaxial MEMS LiDAR design to increase the detection range, in which two MEMS mirrors were synchronous to increase the receiving aperture [13,14]. As shown in Figure 1a, an ultra-large-aperture MEMS mirror2 is employed in the semi-coaxial MEMS LiDAR, and the upper half of MEMS mirror2 is used for emitting light and the lower half is used for receiving light. Due to the difficulties in design and fabrication of an ultra-large-aperture MEMS mirror, the method is proposed to increase the receiving aperture of MEMS LiDAR by synchronizing the movement of the MEMS mirror3 and MEMS mirror4 to replace the MEMS mirror2, as shown in Figure 1b. In order to achieve the synchronization of two MEMS mirrors, it is essential to focus on the research of the motion model and control methodology of two MEMS mirrors.

By means of voltage compensation and phase adjustment, the rapid synchronization of two comb-actuated electrostatic MEMS mirrors was achieved in our early study [14]. However, the electrostatic MEMS mirror is typically small in size due to being controlled by electrostatic force, which makes it difficult to achieve a large-aperture reception [15]. By contrast, the electromagnetic MEMS mirror is more capable of achieving a larger aperture [16]. In this paper, we further investigate the motion characteristics of the electromagnetic MEMS mirrors and develop a synchronous motion model driven by electromagnetic force. The simulation parameters of electromagnetic MEMS mirrors are deeply analyzed, and the amplitude-frequency characteristics, phase-frequency characteristics, and Bode plots of two electromagnetic MEMS mirrors are obtained. A synchronization experiment on two electromagnetic MEMS mirrors is carried out, achieving the synchronized motion through current compensation and phase adjustment. The entire synchronization procedure is described in detail, and the simulation and experimental results prove the feasibility of synchronizing large-aperture electromagnetic MEMS mirrors to increase the detection range of MEMS LiDAR.

The rest of this paper is organized as follows: Section 2 gives the synchronous motion model of two electromagnetic MEMS mirrors. Section 3 presents a detailed analysis of the simulation parameters for the electromagnetic MEMS mirrors, outlining the complete synchronization procedure. In Section 4, the experiments are carried out to verify the feasibility of the large-aperture electromagnetic MEMS mirrors. In Section 5 and Section 6, the discussion and conclusions are presented.

## 2. Modeling the Motion of the Electromagnetic MEMS Mirror

**Figure 2:**
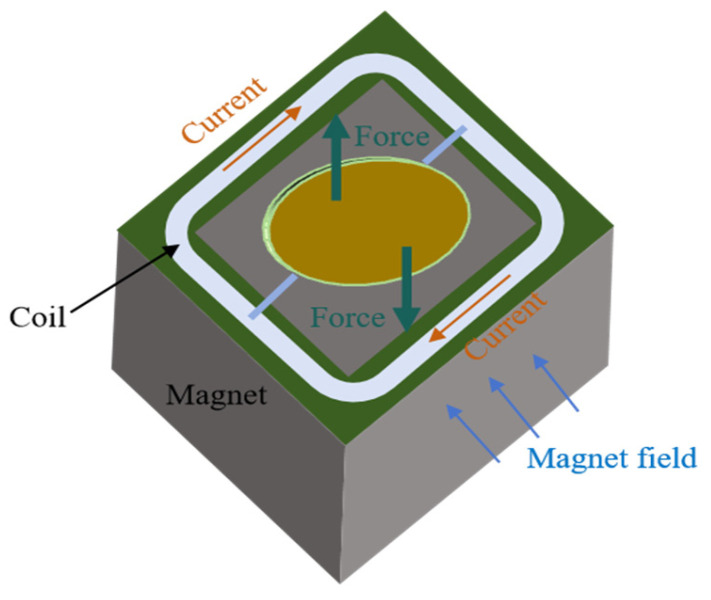
Structural design of the electromagnetic MEMS mirror.

The design principle of the electromagnetic MEMS mirror involves attaching magnetic materials near the MEMS mirror. The interaction between the current and magnetic materials creates a magnetic field, which in turn generates an electromagnetic force causing the MEMS mirror to undergo torsional movement [17,18]. Typically, coils are strategically placed around the MEMS mirror which is situated within a permanent magnetic field as shown in Figure 2. When current flows through the coils, the interaction between the current and permanent magnetic field generates Lorentz force, which drives the MEMS mirror to deflect through a specific structure. In contrast to the electrostatic MEMS mirror, the electromagnetic MEMS mirror operates with a lower driving voltage, exhibits linear force characteristics, and is more capable of obtaining a large mirror surface area.

In order to establish a motion model for the MEMS mirror, it is necessary to research the force acting on it and the motion generated as a result of the force. When a one-dimensional MEMS mirror is actuated by a driving signal, it typically behaves similarly to a mass block rotating about an axis under the influence of a driving torque. The MEMS mirror motion model can be represented by a second-order oscillatory system as shown in Equation (1) [19,20,21].

(1) $$J\frac{d^{2}\theta }{dt^{2}}+C\frac{d\theta }{dt}+K\theta =M$$

Here, J is the moment of inertia, C is the damping coefficient, K is the elasticity coefficient, θ is the motion angle of the MEMS mirror, M is the driving torque, and t is time.

When the MEMS mirror moves at a small angle, the torsional resonance system formed by the motion can be considered linear. The response of an ideal linear system to a nonlinear excitation can be thought of as a superposition of all the frequency responses that make up the excitation wave [21]. When the MEMS mirror is subjected to a sinusoidal excitation, the torque it experiences can be represented by Equation (2), its response amplitude can be expressed as Equation (3), and its response phase can be expressed as Equation (4).

(2) $$M=M_{0}sin\left(\omega_{d}\right)$$

(3) $$\theta_{0}=\frac{M_{0}}{\sqrt{{\left(K-J\omega_{d}^{2}\right)}^{2}+C^{2}\omega_{d}^{2}}}$$

(4) $$\varphi =tan^{-1}\left(\frac{C\omega_{d}}{K-J\omega_{d}^{2}}\right)$$

Here, $M_{0}$ is the amplitude of the driving torque, $\omega_{d}$ is the angular frequency, $\theta_{0}$ is the motion amplitude, and φ is the motion phase of MEMS mirror.

As shown in Equations (1)–(4), the motion characteristics of the MEMS mirror are significantly influenced by the manufacturing process and the parameters of the driving signal, including its amplitude, frequency, and phase. In the case of the electromagnetic MEMS mirror, the driving torque is determined by the total magnetic torque produced by the driving coil, as represented by Equation (5).

(5) $$M=M_{m}\times B$$

Here, $M_{m}$ is the total magnetic torque generated by all the driving coils and B is the magnetic induction intensity.

In Equation (5), the total magnetic torque $M_{m}$ is primarily determined by the amplitude of the driving current. Therefore, the driving torque of the electromagnetic MEMS mirror is directly proportional to its driving current. To simplify the simulation model, the driving coil is considered a first-order linear differential circuit, and its input voltage can be expressed by Equation (6). Furthermore, the driving torque of the electromagnetic MEMS mirror is a linear gain of its driving current, which can be expressed by Equation (7). The transfer function for the driving torque is presented in Equation (8).

(6) $$U_{r}=L_{m}\frac{dI_{m}}{dt}+R_{m}I_{m}$$

(7) $$M=I_{m}\cdot C_{m}$$

(8) $$G_{M}\left(s\right)=\frac{C_{m}}{L_{m}s+R_{m}}$$

Here, $U_{r}$ is the input voltage of the driving coil, $G_{M}\left(s\right)$ is the transfer function for the driving torque, $C_{m}$ is a constant, $L_{m}$ is the equivalent inductance of the driving coil, and $R_{m}$ is the equivalent resistance of the driving coil.

According to Equation (1), the transfer function for the motion amplitude of the electromagnetic MEMS mirror can be expressed by Equation (9). Eventually, the transfer function for the motion of the electromagnetic MEMS mirror is obtained as shown in Equation (10).

(9) $$G_{\theta }\left(s\right)=\frac{1}{Js^{2}+Cs+K}$$

(10) $$G\left(s\right)=G_{M}\left(s\right)\cdot G_{\theta }\left(s\right)=\frac{C_{m}}{\left(Js^{2}+Cs+K\right)\left(L_{m}s+R_{m}\right)}$$

Here, $G_{\theta }\left(s\right)$ is the transfer function of motion amplitude and $G\left(s\right)$ is the transfer function for the motion of the electromagnetic MEMS mirror.

Based on Equations (1)–(10), the motion model of the electromagnetic MEMS mirror is established, which can be used to simulate and obtain the amplitude-frequency and phase-frequency characteristics of the electromagnetic MEMS mirror. It should be noted that the simulated value of the moment of inertia J can be obtained from the structural design parameters of the MEMS mirror. Nevertheless, the variations in manufacturing processes can lead to differences in the actual moment of inertia among different MEMS mirrors.

## 3. Simulation of the Synchronous Motion

To further investigate the synchronization method of the large-aperture electromagnetic MEMS mirrors, the simulation process is carried out as follows. According to Equations (1)–(10), the moment of inertia J can be obtained from the structural design parameters, the damping coefficient C can be calculated by Equation (11) and the elasticity coefficient K can be calculated by Equation (12).

(11) $$K={\left(2\pi f_{m}\right)}^{2}J$$

(12) $$C=\frac{\sqrt{KJ}}{Q}$$

Here, Q is the quality factor of the MEMS mirror and $f_{m}$ is the resonant frequency of the MEMS mirror.

The resonant frequency of the electromagnetic MEMS mirrors designed in this paper is approximately 220 Hz. The equivalent inductance $L_{m}$ and the equivalent resistance $R_{m}$ of the driving coil can be measured by a multimeter. The characteristic parameters of the electromagnetic MEMS mirror $M_{1}$ and the characteristic parameters of the electromagnetic MEMS mirror $M_{2}$ are shown in Table 1.

**Figure 3:**
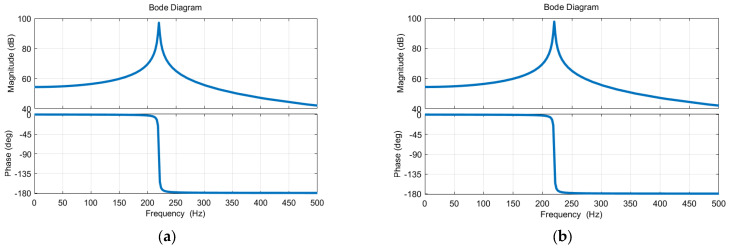
(**a**) Simulation Bode plot of the electromagnetic MEMS mirror $M_{1}$; (**b**) Simulation Bode plot of the electromagnetic MEMS mirror $M_{2}$.

Based on the above characteristic parameters, the transfer functions for the motions of two MEMS mirrors are simulated, and the resulting simulation Bode plots are shown in Figure 3. The analysis indicates that the gain responses of the two MEMS mirrors are very similar, with both exhibiting resonant frequencies of approximately 220 Hz. The phase-frequency responses of the two MEMS mirrors exhibit a consistent pattern, with both experiencing substantial phase shifts in the frequency range of 200 Hz–250 Hz.

**Figure 4:**
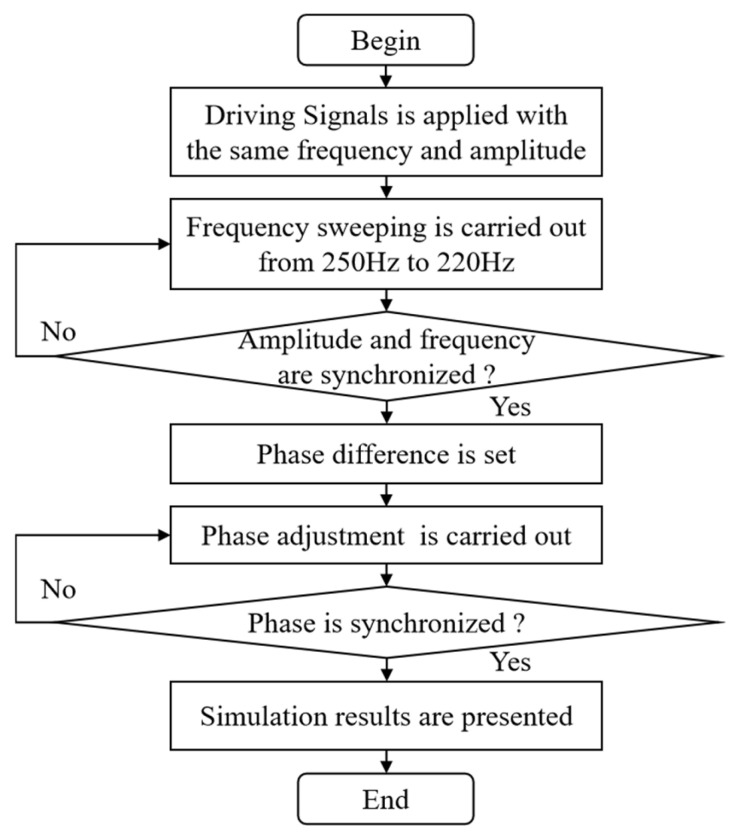
Simulation process for the synchronous motion.

From the Bode plots of the two electromagnetic MEMS mirrors, it can be seen that there are differences in their amplitude-frequency and phase-frequency characteristics. To achieve a synchronous motion for the two MEMS mirrors, it is necessary to synchronize their motion frequency, amplitude, and phase. The simulation process for the synchronous motion is shown in Figure 4. At first, two sine drive signals with the same frequency and amplitude are applied to the two MEMS mirrors. Secondly, a frequency sweep from 250 Hz to 220 Hz is carried out to change the driving torque of the MEMS mirror. Thirdly, real-time monitoring is conducted to ascertain whether the motion frequency and amplitude of the two electromagnetic MEMS mirrors have achieved synchronization. Fourthly, to simulate the motion phase difference between the two MEMS mirrors, a certain phase difference is set for the drive signals. Next, a phase adjustment of the driving signal is carried out and real-time monitoring is conducted to ascertain whether the motion phase of the two electromagnetic MEMS mirrors has achieved synchronization. Finally, the simulation results are presented.

**Figure 5:**
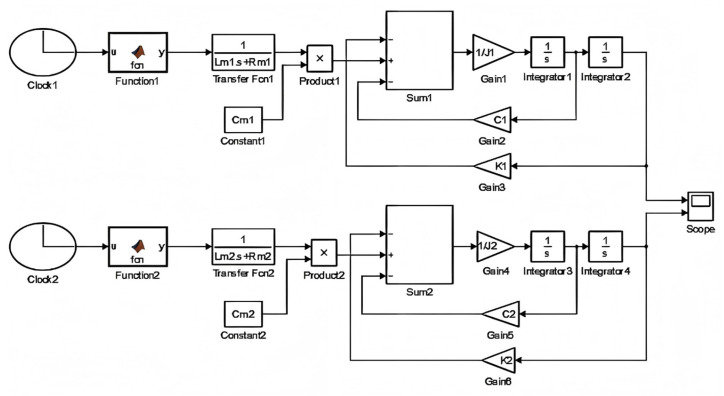
Simulation model in MATLAB Simulink.

According to Equation (10), the entire simulation process for the synchronous motion of the two electromagnetic MEMS mirrors is conducted in the MATLAB R2014a Simulink as shown in Figure 5. The upper part of the simulation block diagram constructs the motion model of the MEMS mirror $M_{1}$, and the lower part for the MEMS mirror $M_{2}$. In the simulation model, the driving signals for the two MEMS mirrors are generated by a custom function. As shown in Figure 5, Function1 and Function2 are used to generate the driving signals for MEMS mirrors $M_{1}$ and $M_{2}$. Moreover, the frequency of the driving signal is varied from 250 Hz to 220 Hz to achieve a frequency sweep. The initial phase of the two driving signals is set to 0° and 90°, respectively, to simulate the phase difference in the motion of the two MEMS mirrors. Transfer Fcn1 and Transfer Fcn2 are used to construct the transfer function for the driving torque. Characteristic parameters $L_{m}$, $R_{m}$, $C_{m}$, J, C, and K of the two MEMS mirrors can be obtained from Table 1. The different motion states of the two electromagnetic MEMS mirrors can be observed in real time by the scope module.

**Figure 6:**
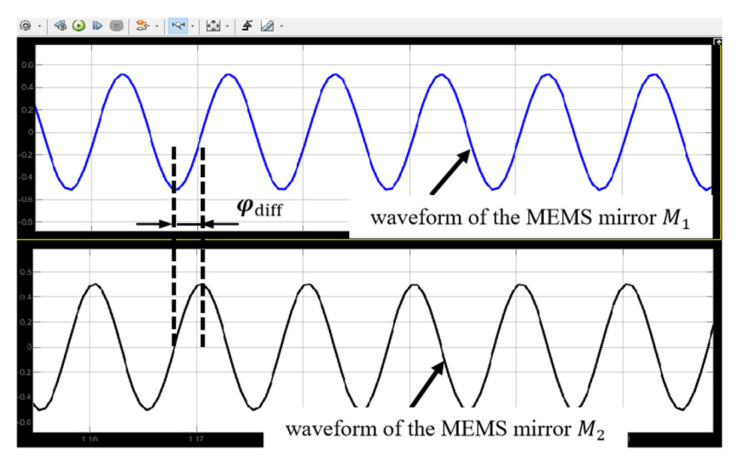
Simulation results at the initiation of the synchronization.

Figure 6 shows the simulation results of the initial motion states of the two electromagnetic MEMS mirrors. As can be seen, the two electromagnetic MEMS mirrors have the same motion frequency and amplitude, but there is a difference in motion phase, which means the motion characteristics of the electromagnetic MEMS mirrors are consistent with the characteristics of their driving signals.

**Figure 7:**
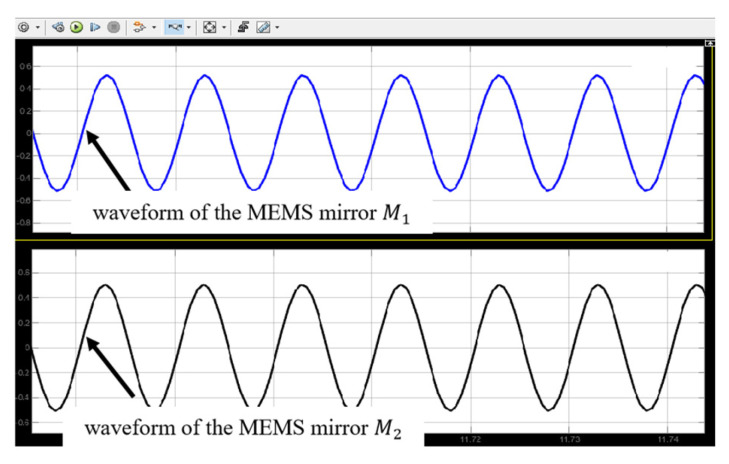
Simulation results when the synchronization is achieved.

Figure 7 shows the simulation results when the motion synchronization of the two electromagnetic MEMS mirrors is completed. It can be seen that by adjusting the frequency and phase of the driving signals, the synchronization of the motion frequency, amplitude, and phase of the two electromagnetic MEMS mirrors is achieved.

Through the analysis of the above simulation results of the MEMS mirror synchronization, it can be known that by adjusting the frequency, amplitude, and phase of the drive signals, the driving torque of the MEMS mirror will change accordingly. Then the motion responses of the MEMS mirrors alter and, ultimately, the motion synchronization of the two electromagnetic MEMS mirrors can be realized. The simulation results serve as a robust foundation for the subsequent experimental research on the synchronized motion of MEMS mirrors.

## 4. Experimental Section

**Figure 8:**
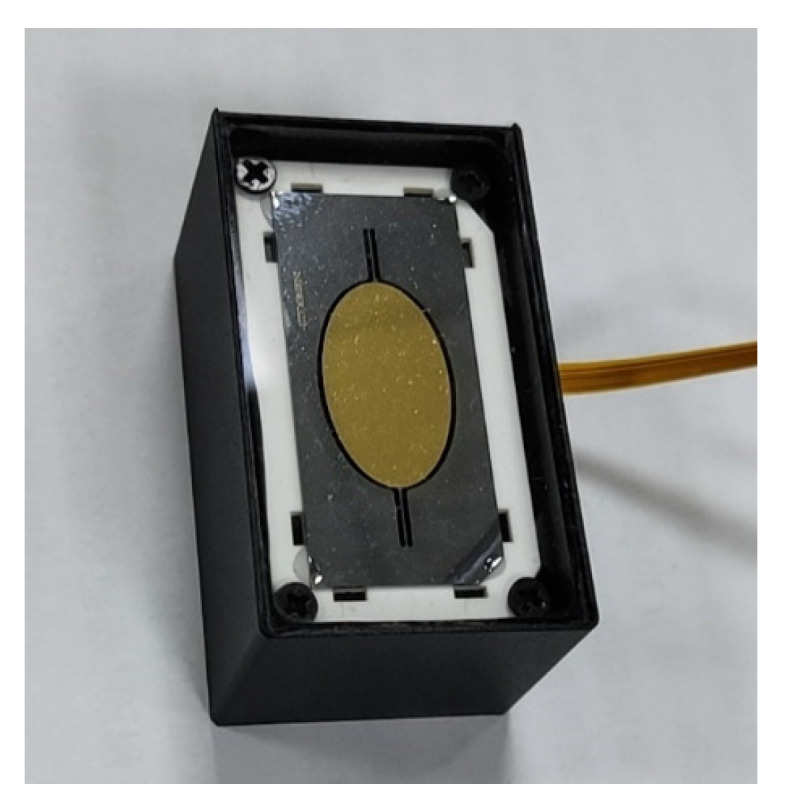
Electromagnetic MEMS mirror module.

To verify the feasibility of synchronizing two large-aperture electromagnetic MEMS mirrors, the experiment utilized two electromagnetic MEMS mirror modules that were independently developed by our research group. The MEMS mirror modules are integrated with the MEMS mirrors and their feedback control system, and can monitor the motion states of the MEMS mirrors in real-time. Figure 8 shows the electromagnetic MEMS mirror module and Table 2 shows the specifications of the two electromagnetic MEMS mirrors.

As shown in Table 2, the two electromagnetic MEMS mirrors have a large aperture of 60${\pi \;\mathrm{mm}}^{2}$, and the resonant frequencies are 220.6 Hz and 221.1 Hz. Meanwhile, there is a difference in the phase between the two MEMS mirrors. It should be noted that the phase difference in Table 2 represents the difference between the driving signal and the motion cycle signal provided by the feedback system.

**Figure 9:**
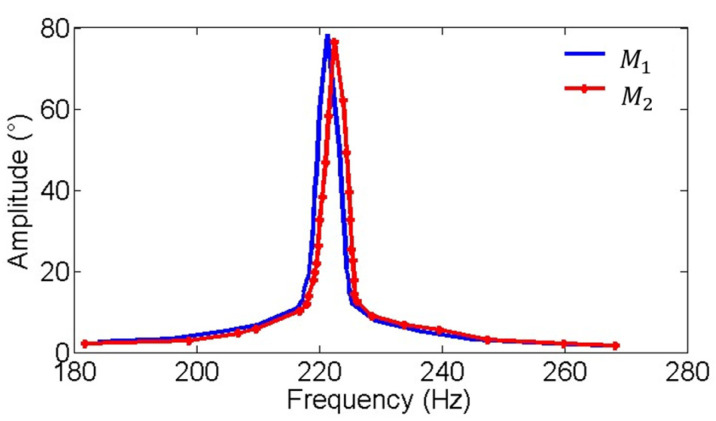
Amplitude-frequency characteristic of the MEMS mirror $M_{1}$ and the MEMS mirror $M_{2}$.

**Figure 10:**
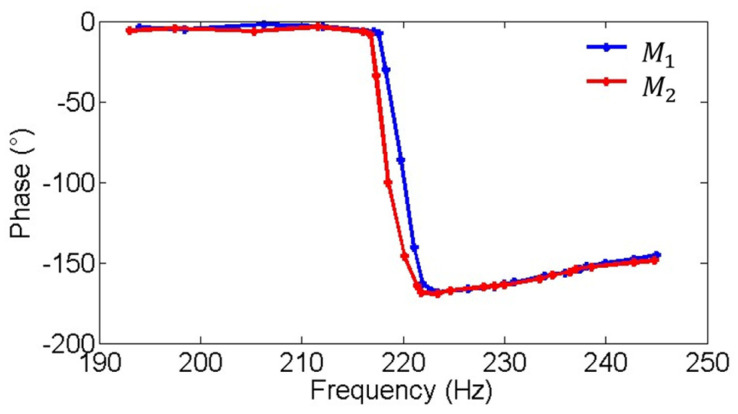
Phase-frequency characteristic of the MEMS mirror $M_{1}$ and the MEMS mirror $M_{2}$.

**Figure 11:**
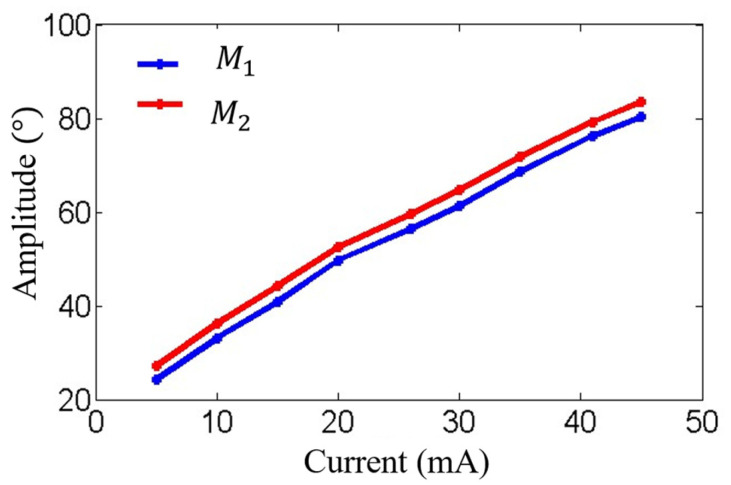
Variation of amplitude with driving current intensity of the MEMS mirrors.

Figure 9 and Figure 10 show the amplitude-frequency and phase-frequency characteristics of the two electromagnetic MEMS mirrors. It can be observed that the two MEMS mirrors demonstrate highly consistent amplitude-frequency characteristics, with both achieving their peak amplitude at approximately 220 Hz. Additionally, a phase difference exists between the two MEMS mirrors at the resonant frequency of 220 Hz. Figure 11 illustrates the process of how the vibration amplitude of the MEMS mirror changes with the intensity of the driving current, which can be used to adjust the current intensity of the driving signal to compensate for the amplitude difference between the two electromagnetic MEMS mirrors.

**Figure 12:**
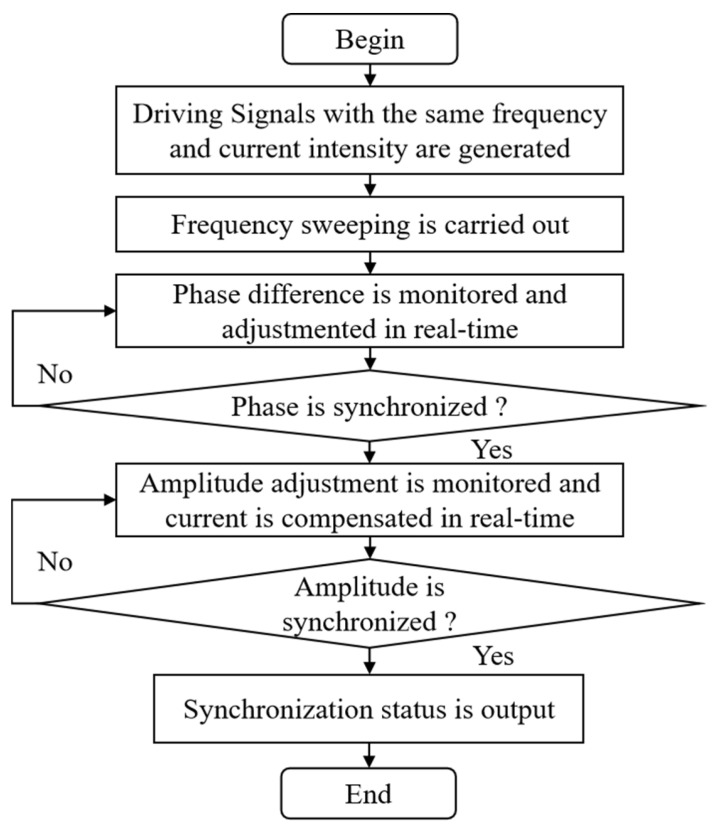
Experimental procedure for the synchronous motion.

Based on the above motion model and simulation results, the procedure of the motion synchronization experiment of the two electromagnetic MEMS mirrors is shown in Figure 12. At first, two driving signals with the same frequency and current intensity generated by the Direct Digital Synthesizer (DDS) are used to activate the MEMS mirrors. Secondly, the frequency of the driving signal is progressively decreased from a higher to a lower value, thereby achieving a frequency sweep. Thirdly, the feedback control system monitors the phase difference between the two MEMS mirrors in real time, continuously adjusting the phases of the driving signals to reduce the phase difference. Fourthly, a judgment is made on whether the phases of the two electromagnetic MEMS mirrors are synchronized. Next, the amplitude difference between the two MEMS mirrors is monitored in real time, and the current is continuously adjusted to compensate for it. Then, a judgment is made again on whether the amplitudes of the two electromagnetic MEMS mirrors are synchronized. Finally, the synchronization status of the two electromagnetic MEMS mirrors is output.

**Figure 13:**
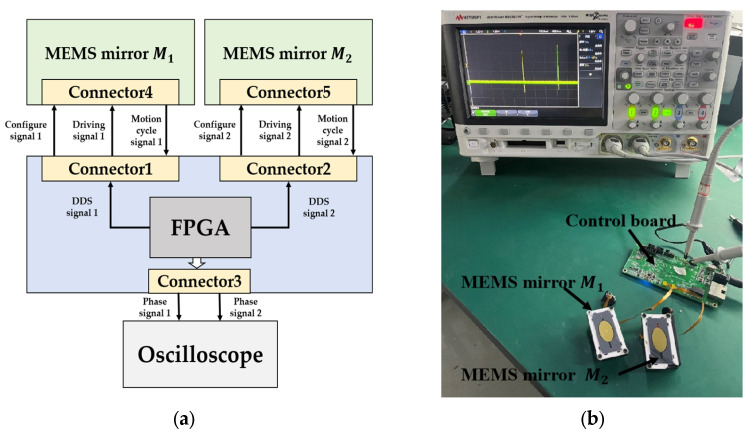
(**a**) Schematic of the FPGA control system; (**b**) Picture of the experiment for the synchronous motion.

In the synchronization process of the electromagnetic MEMS mirrors, the MEMS mirror modules are connected to the main control board. After power is applied, the FPGA on the motherboard generates driving signals based on the Direct Digital Synthesizer and then outputs the driving signals to the MEMS mirrors. A frequency sweep is carried out from 250 Hz to 220 Hz. The MEMS mirror modules provide the motion cycle signals of the electromagnetic MEMS mirrors to the FPGA to detect the phase difference between the two MEMS mirrors. During the frequency sweep, the phase difference between the MEMS mirrors is gradually reduced by adjusting the phase of the DDS driving signal. The phase adjustment is continuous throughout the entire frequency sweep process, and the phase synchronization error must ultimately be controlled within the allowable range. After the frequency sweep is completed, the current intensity of the driving signal is adjusted in real time to achieve amplitude synchronization of the MEMS mirrors. Finally, the synchronization is completed, both MEMS mirrors have an optical scanning angle of 60°, a vibration frequency of 220 Hz, and a phase synchronization error within 2.8 µs, which meets the phase synchronization error requirement of 0.2° angular resolution. The entire synchronization process is completed within 10 s. The control system and experimental setup are shown in Figure 13.

## 5. Discussion

This study proposes a synchronization approach for two large-aperture electromagnetic MEMS mirrors, and a synchronous motion simulation model is constructed. Compared to other solutions, this method exhibits advantages in achieving a large aperture and long detection range for MEMS LiDAR. Nevertheless, this study has certain limitations. The construction of the motion model is rather idealized and simplified, with many factors yet to be considered, such as the noise signals and control accuracy. Moreover, the synchronization results obtained from the experiment show a certain difference from those of the simulation. Future research will optimize the simulation model and conduct extensive sample experiments to improve the system’s stability and accuracy. In addition, we will pay attention to relevant industrial standards to enhance the feasibility of commercializing this research with the aim of providing technical support for the advanced MEMS LiDAR industry.

## 6. Conclusions

In this paper, the synchronization method for two large-aperture electromagnetic MEMS mirrors is investigated. A synchronous motion model of two electromagnetic MEMS mirrors is constructed, which thoroughly analyzes the relationship between the motion state of the electromagnetic MEMS mirror and its amplitude-frequency characteristics, phase-frequency characteristics, and structural composition. A simulation is carried out in a MATLAB simulation and the synchronous motion simulation process of two electromagnetic MEMS mirrors is revealed in detail. An experiment is set up by employing two electromagnetic MEMS mirror modules which integrate the MEMS mirrors and their feedback control system. Finally, two electromagnetic MEMS mirrors are synchronous with an aperture of 60${\pi \;\mathrm{mm}}^{2}$, field of view (FoV) of 60°, and scanning frequency of 220 Hz. The experimental results verify the feasibility of synchronizing large-aperture electromagnetic MEMS mirrors and provide a promising potential to achieve a large-aperture and large-detection-range MEMS-based LiDAR system.

## Figures and Tables

**Table 1:** Characteristic parameters of the electromagnetic MEMS mirrors for simulation.

Parameters	$M_{1}$	$M_{2}$
J $\left(g\cdot {\mathrm{mm}}^{2}\right)$	0.99960132	0.99970251
C	$1.00490885\times 10^{-8}$	$9.42197418\times 10^{-9}$
K	0.00190999	0.00191019
$L_{m}\;(\mathrm{mH})$	3	2.8
$R_{m}\;(\Omega )$	6.2	6.4
$C_{m}$	0.00153451	0.0015422

**Table 2:** Specifications of the electromagnetic MEMS mirrors.

MEMS Mirror	($\mathrm{Aperture}\;mm^{2}$)	Resonant Frequency (Hz)	Optical Amplitude (°)	Phase Difference (°)
$M_{1}$	60π	220.6	60	−132.4
$M_{2}$	60π	221.1	60	−150.3

## Data Availability

The original contributions presented in this study are included in the article. Further inquiries can be directed to the corresponding author.
